# Mutational spectrum of *SMPD1* gene in Pakistani Niemann-Pick disease patients

**DOI:** 10.12669/pjms.36.3.467

**Published:** 2020

**Authors:** Huma Arshad Cheema, Iqra Ghulam Rasool, Muhammad Nadeem Anjum, Muhammad Yasir Zahoor

**Affiliations:** 1 Dr. Huma Arshad Cheema, MBBS, MCPS, DPGN. Department of Pediatric Gastroenterology and Hepatology, The Children’s Hospital & The Institute for Child Health, Lahore, Pakistan; 2 Iqra Ghulam Rasool, M.Phil. Institute of Biochemistry & Biotechnology, University of Veterinary & Animal Sciences, Lahore, Pakistan; 3 Dr. Muhammad Nadeem Anjum, MBBS, FCPS. Department of Pediatric Gastroenterology and Hepatology, The Children’s Hospital & The Institute for Child Health, Lahore, Pakistan; 4 Dr. Muhammad Yasir Zahoor, PhD. Institute of Biochemistry & Biotechnology, University of Veterinary & Animal Sciences, Lahore, Pakistan

**Keywords:** Acid sphingomyelinase, Mutations, Niemann Pick disease, *SMPD1*

## Abstract

**Objective:**

Genetic variation analysis of rare autosomal recessive Niemann-Pick disease (NPD) Pakistani patients.

**Methods:**

We sequenced the *SMPD1* gene including its all coding and flanking regions in seven unrelated sporadic patients suffering from Niemann-Pick disease through targeted exome sequencing. Genetic variants mapping and their protein predictions were evaluated using different bioinformatics tools and clinical phenotypes were correlated. The study was conducted from January 2018 to March 2019 at The Children’s Hospital Lahore.

**Results:**

We have mapped five different mutations in *SMPD1* gene of enrolled patients with a novel homozygous missense variant (c.1718G>C) (p.Trp573Ser) in one patient. A missense mutation (c.1267C>T) (p.His423Tyr) has been identified in three unrelated patients. A nonsense mutation (c.1327C>T) (p.Arg443Term) and one missense mutation (c.1493G>A) (p.Arg498His) mapped in one patient each. A compound heterozygous mutation has been mapped in one patient (c.740G>A) (p.Gly247Asp); (c.1493G>A) (p.Arg498His). Pathogenic effect of novel variant has been predicted through in-silico analysis and has not been reported in general overall population in the globe.

**Conclusion:**

This is the first report of genetic demographic assessment of Niemann-Pick disease in Pakistan. The mapped mutations would be helpful to build a disease variants algorithm of Pakistani population. This will be used for determining disease clinical magnitude along with provision of genetic screening services in affected families.

## INTRODUCTION

Niemann-Pick disease (NPD) OMIM # 607608; is caused by the deficiency or inactivity of acid sphingomyelinase enzyme (ASM) EC 3.1.4.12, encoded by *SMPD1* gene. The disease is associated with mutations in ASM encoding gene *SMPD1*, resulting in sphingomyelin accumulation in reticuloendothelial cells and hepatocytes.^1^ Clinically, NPD patients usually present with enlargement of body organs such as liver and spleen (hepatosplenomegaly), fail to weight gaining and growth in a usual way (failure to thrive), progressive loss of mental abilities, loss of muscle movement, eye abnormality called a cherry-red spot and recurrent lung infections. NPD can be classified into two common types: NPD type A and NPD type B, based on the onset of the disease, severity of symptoms and type of organs affected both caused by variation in *SMPD1* gene. The NPD types A and B has been reported for affecting 1 in 250,000 individuals.^2^,^3^ NPD type A affects in early infancy with organomegaly and then a neurodegenerative course manifested around three months of age leading to demise by three years while, with NPD type B patients often survive into adulthood correlate with hepatosplenomegaly and respiratory complications and have less or no CNS involvement.^4^,^5^

Acid sphingomyelinase is a lipid hydrolase that catalyzes sphingomyelin (SM) to phosphorylcholine and ceramide. Breakdown of SM is carried out by acid sphingomyelinases (ASMases), which cut the phosphodiester bond of SM producing the phosphorylcholine head group to generate ceramide. Deficiency or inactivity of ASMase leads to accumulation of SM resulting in Niemann-Pick disease (OMIM; NPD-A: 257200, NPD-B: 607616).^2^,^3^

The *SMPD1* gene is positioned on chromosome 11p15.4-p15.1 and containing 6 exons spans approximately ~6 kilobases and encodes a protein of 631 amino acids.^6^ At present more than 133 missense and nonsense mutations have been reported worldwide in *SMPD1* gene related to Niemann-Pick disease spreading coding domains of the gene (Human Gene Mutation Database accessed on 11-02-2019; http://www.hgmd.cf.ac.uk/ac/all.php).

Autosomal recessive disorders are present more frequently in Pakistani populations due to high rate of consanguinity. Almost 65% of the marriages are consanguineous in Pakistan among these first cousins’ marriages accounts more than 74%.^7^ We have performed genetic investigation for causative variants of ND disease and mapped five mutations in *SMPD1* gene among patients from segregating the NPD disease and have consanguineous unions.

## METHODS

### Enrollment of Patients

Total seven sporadic patients having Niemann-Pick disease (named PKNP 1-7) were enrolled with informed consent for genetic evaluations through collaboration of the Children Hospital Lahore. The patients were enrolled based on their clinical presentation, medical history and biochemical findings. Clinical data includes onset of the disease, hepatosplenomegaly, progressive neurodegenerative involvement, failure to thrive along with presence of reduced acid sphingomyelinase activity in fibroblasts, lymphoblasts or in peripheral white blood cells. Patients belong to different provinces as those coming to the Children Hospital Lahore being facilitated for cure of these disease. Blood samples were obtained for molecular analysis of the *SMPD1* gene. NPC1 & NPC2 were not screened as the patients were not manifesting related symptoms and variants mapped in *SMPD1*. The study was conducted from January 2018 to March 2019 at The Children’s Hospital Lahore.

### DNA extraction and targeted Sequencing

DNA was isolated using standard protocols from the blood samples of enrolled patients. The extracted DNA was stored under appropriate conditions for future analysis after analyzing quantity and quality using spectrophotometric analysis. To identify the underlying pathogenic mutation causing the disease, DNA of the affected probands were subjected to targeted exome sequencing of all coding exons and flanking intronic regions of *SMPD1* gene (HiSeq 2500 System, Illumina, Archimed Life, Vienna, Austria). Data were analyzed using various bioinformatics tools for variation analysis and protein prediction.

## RESULTS

**Fig.1:**
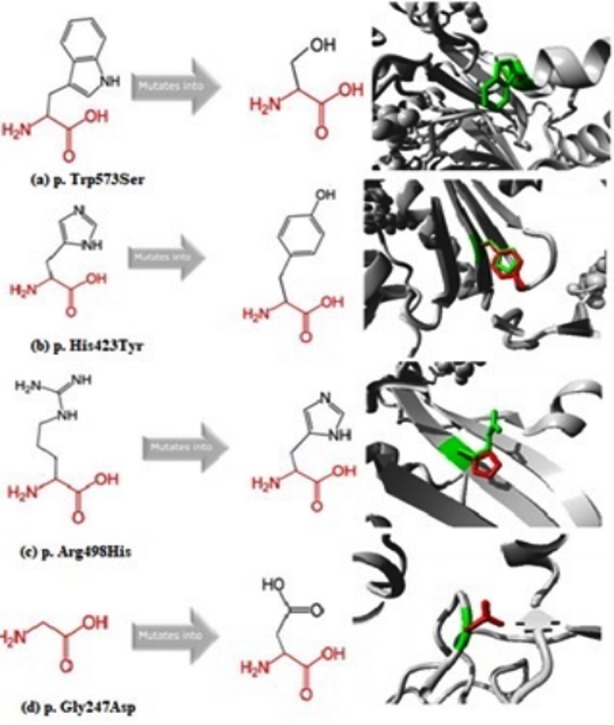
Description of wild type and mutant amino acids and structural changes (green color depicts wild type whereas red color showed mutant type residue).

We have identified five different disease causing mutations of *SMPD1* gene in Niemann-Pick disease unrelated patients from consanguineous families. A novel variant (c.1718G>C) (p.Trp573Ser) has been identified in one patient in homozygous form. A missense mutation (c.1267C>T) (p.His423Tyr) identified in three different patients in recessive form. A nonsense mutation (c.1327C>T) (p.Arg443Term) has been illustrated in one patient. Another homozygous missense variant (c.1493G>A) (p.Arg498His) has been mapped in another patient. While a compound heterozygous mutation has been mapped in one patient (c.740G>A) (p. Gly247Asp); (c.1493G>A) (p. Arg498His). The brief medical history of the patients has been described in Table-I. The structural descriptions of variants have been described in Fig.1.

**Table-I:** Mutations in SMPD1 identified in Pakistani Patients with NPD.

SMPD1 Mutation	Amino Acid Change	Patient ID	Gender	Age at enrollment	Type of Mutation	Exons	gnomAD Frequency	ExAC	Consanguinity	Clinical Symptoms	Area
c. 1718G>C	p. Trp573Ser	PKNP1	Male	12 months	Missense	6	0	0	Yes	Abdominal Distention Recurrent Chest infection PCV transfusion for once	Mianwali
c. 1267C>T	p. His423Tyr	PKNP4	Male	13 months	Missense	3	(0/1/143266) 6.98e-6	0	Yes	Abdominal Distention Respiratory tract infection	KPK
		PKNP6	Female	3 years					Yes	Mild to Moderate Developmental Delay Abdominal Distention Malena 4 times PCV Transfusion Recurrent Respiratory tract infection	KPK, Afghan Border
		PKNP7	Female	21 months					Yes	Moderate Developmental Delay Progressive Pallor Abdominal Distension PCV transfusion twice Recurrent Chest infection Underweight Stunted	Sindh
c. 1327C>T	p. Arg443Term	PKNP2	Female	19 months	Nonsense	3	(0/6/143204) 4.19e-5	8.25e-6	Yes	Progressive Pallor followed by abdominal distension Recurrent respiratory tract infection Mild to Moderate Developmental Delay Anicteric Abdomen distended	Hyderabad Sindh
c. 1493G>A	p.Arg498His	PKNP5	Female	13 months	Missense	4	0	0	Yes	Mild Development Delay Abdominal Distention Recurrent Respiratory tract infection Failure to thrive	Baluchistan
c.740G>A; 1493G>A	p. Gly247Asp; Arg498His	PKNP3	Female	17 months	Missense	2, 4	0,0	0,0	Yes	Progressive Abdominal Distention 3 times PCV Transfusion Recurrent Respiratory tract infection	Lahore

## DISCUSSION

Acid sphingomyelinase (ASM) is an enzyme that hydrolyze lipids and its deficiency or low activity lead to Niemann-Pick disease types A and B.^8^ ASM catalyzes the hydrolytic cleavage of sphingomyelin in lysosomes, producing phosphocholine and ceramide.^9^ Enzyme inactivity cause the progressive accumulation of sphingomyelin and other lipids in target tissues, and is clinically also termed as ASM deficiency disease (ASMD) responsible for the clinical spectrum of NPD-A or NPD-B metabolic phenotypes.^10^ The pattern of inheritance of ASMD is autosomal recessive, and associated with variants of *SMPD1*. Carrier frequency of *SMPD1* gene variants varies among populations and some of them restricted to certain families and distinct groups.^9^-^12^ The inborn error of metabolism had mostly recessive pattern of inheritance and have increased likelihood to prevail in consanguineous population.^13^-^15^ Here, we demonstrate that the genetic variants of *SMPD1* gene is Pakistani NPD patients along with carriers influences on the disease phenotype.

### c. 1718G>C mutation

We have detected a novel homozygous variant c.1718G>C (p. Trp573Ser) in one patient (PKNP1). The patient was presented at age of one year with progressive abdominal distension and vomiting and belongs to Mianwaali, Punjab Pakistan. The novel c.1718G>C variant produces a replacement of Tryptophan (Try) with Serine (Ser) at position 573. Wild type residue (tryptophan) contains positive charge while mutated residue (serine) contain hydroxyl group which indicates the difference in their hydrophobicity and size (Fig.1a).^16^ The position of amino acid is located in the core of the protein which predicted that mutated residue will cause loss of hydrophobic interaction and places an unoccupied space in the core of protein.^17^ The loss of interaction between these domains by the mutation reduces protein catalytic activity. A different variant at 573 position with termination codon has been reported in Italian patient.^18^ This novel variant was not found in Human Gene Mutation Database (http://www.hgmd.cf.ac.uk/ac/index.php) accessed on 12-02-2019, supporting the notion that this is not a reported variant previously. Furthermore, bioinformatics tools predicted this variant (p. W573S) as “damaging” the protein in structure and function as well.

### c. 1267C>T mutation

The c.1267C>T (p. His423Tyr) mutation was identified in three unrelated probands/patients (named PKNP4, 6, 7). PKNP4 is a 13 months old child, belongs to KPK province, Pakistan, presented with abdominal distention (liver: 7cm; spleen: 12cm), loose motion and worse chest infection. PKNP6 is three years old baby girl migrated from Kabul, Afghanistan in KPK. She had mild to moderate developmental delay, abdominal distention (liver: 13cm; spleen: 20cm) and history of PCV (packed red blood cells) transfusion. She is also having recurrent respiratory tract infections. PKNP7 is a 21 months old child, belongs to Interior Sindh, having moderate developmental delay, progressive pallor and abdominal distension (liver: 6cm; spleen: 14cm) since the age six months. She was transfused PCV twice during infancy and has history of recurrent chest infections and one-time admission due to RTI (Respiratory tract Infections). General physical examination showed that she is pale, underweight and stunted.

Mutation c.1267C>T depicted that wild type residue (Histidine) has less hydrophobicity than mutated residue (Tyrosine) and is larger in size than wild type. The difference in size of mutant residue and wild-type displaces the position so that the new residue is not at a place to make the same hydrogen bond as the wild-type residue fixes normally. The wild-type residue was buried in the core of the protein and new variant mutation disrupts correct folding of protein through loss of hydrogen bonds in the core of the protein (Fig.1b). Furthermore, various bioinformatics tools also have been predicting a damaging mutation. The c.1267C>T (p. His423Tyr) mutation was reported in Saudi Arabian patients, which accounts for the ~85% of the disease allele in those patients.^19^ Here, we report this mutation in three unrelated Pakistani patients and can be predicted that this disease allele may have more penetration in our population and can be among major screening variants in affected families.

### c. 1327C>T mutation

A mutation with termination codon (c. 1327C>T) (p. Arg443Term) was detected in one patient (PKNP2). PKNP2 is a 19 months old child from Hyderabad, presented with progressive pallor followed by abdominal distension (liver: 5cm; spleen: 14cm) since the age of seven months. He has mild to moderate developmental delay and has history of recurrent respiratory tract infections. On examination, he was found pale and anicteric. No ascites was observed.

Mutation (c. 1327C>T) showed that the wild type residue (Arginine) was replaced by stop codon. This variant forms a premature termination codon at position 443; Arg443Term. This mutation leads to the formation of non-functional protein. The (c.1327C>T) (p.Arg443Term) mutation has been reported in Asian Indian descent of Niemann-Pick disease.^20^

### c. 1493G>A mutation

he (c.1493G>A) (p.Arg498His) missense mutation was mapped in one patient (PKNP5) in homozygous form and heterozygous form in one patient PKNP3. PKNP5 is a two and half years old child who belongs to Baluchistan, Pakistan. She was suffering from mild development delay and showed progressive abdominal distention since age one year. Other symptoms include recurrent respiratory tract infection and failure to thrive.

Mutation (c. 1493G>A) showed that the wild-type residue (Arginine) carries positive charge while the mutant residue (Histidine) has a neutral charge and is smaller in size. Arginine is involved in salt bridge formation with aspartic acid at position 463 so the difference in charge will disturb the ionic interaction made by the arginine (Fig.1c). Mutant residue is located on a significant conserved domain and such variants have highly damaging possessions. The (c. 1493G>A) mutation has been reported in Italian population.^18^

### Compound heterozygous c.740G>A; 1493G>A mutation

One mutations (c.740G>A); was mapped in one patient (PKNP3) in compound heterozygous form with another heterozygous variant (c.1493G>A). PKNP3 is a 17 months old child from Lahore, Pakistan, presented with progressive abdominal distention since age seven months and having history of PCV transfusion. Other symptoms include recurrent respiratory tract infections.

Mutation (c.740G>A) result in replacement of Glycine at position 247 to Aspartic acid (p. Gly247Asp). The wild type residue (Glycine) is neutral and mutant type residue (Aspartic acid) carries negative charge (Fig.1d). The size of mutant type was bigger than wild type. Due to size and charge difference, the mutant residue has not an accurate position to make a bond with other residues. This mutation (c.740G>A); (p. Gly247Asp) has been reported in two Hungarian patients.^21^ In second variant (c.1493G>A); (p. Arg498His), the wild-type residue (Arginine) carries positive charge while the mutant variant (Histidine) has a neutral charge and is smaller than arginine as discussed in previous segment. The (c. 1493G>A) mutation has been reported in Italian population.^18^ The inherited disorders in children including the lysosomal storage disorder in Pakistan need to be focused for molecular studies and the trend has been increasing as it will provide child health care.^22^-^25^

## CONCLUSION

This is the first report for mutation spectrum of *SMPD1* gene in Pakistan contributing to development in genomic variants algorithm of NPD in indigenous population. Molecular analysis for NPD would be helpful for early and accurate diagnosis leading to a proper disease management.

### Authors’ Contribution:

**HAC & MYZ** executed work and are responsible and accountable for the accuracy or integrity of the work.

**MNA** did data collection and patients enrollment.

**IGR & MYZ** performed data analysis and manuscript writing.
